# A Current Review of the Uses of Bioelectrical Impedance Analysis and Bioelectrical Impedance Vector Analysis in Acute and Chronic Heart Failure Patients: An Under-valued Resource?

**DOI:** 10.1177/10998004221132838

**Published:** 2022-11-07

**Authors:** Jenjiratchaya Thanapholsart, Ehsan Khan, Geraldine A. Lee

**Affiliations:** 1 Division of Applied Technologies for Clinical Care, Florence Nightingale Faculty of Nursing, Midwifery and Palliative Care, 4616 King’s College London, London, UK; 2 Department of Adult Nursing, Florence Nightingale Faculty of Nursing, Midwifery and Palliative Care, 4616 King’s College London, London, UK

**Keywords:** bioelectrical impedance analysis, bioelectrical impedance vector analysis, BIVA, heart failure

## Abstract

**Background:**

There is a need to detect and prevent fluid overload and malnutrition in heart failure. Bioelectrical impedance analysis and bioelectrical impedance vector analysis are medical instruments that can advance heart failure management by generating values of body composition and body water, assisting clinicians to detect fluid and nutritional status. However, there is a lack of evidence to summarise how they have been used among heart failure patients.

**Method:**

A systematic search was conducted.

**Result:**

Two hundred and four papers were screened. Forty-eight papers were reviewed, and 46 papers were included in this review. The literature shows that bioelectrical impedance analysis and bioelectrical impedance vector analysis were mostly used to assess fluid and nutritional status, together with diagnostic and prognostic values. Contraindication of using BIA and implications for practice are also demonstrated.

**Conclusion:**

The findings suggest that bioelectrical impedance vector analysis is superior to bioelectrical impedance analysis when assessing hydration/nutritional status in heart failure. Assessing a patient using bioelectrical impedance analysis /bioelectrical impedance vector analysis, together with natriuretic peptide -heart failure biomarkers, increases the diagnostic accuracy of heart failure. Further studies are required to examine the cost effectiveness of using these instruments in clinical practice.

## Introduction

Heart failure is commonly associated with fluid overload. An assessment of fluid congestion is crucial in heart failure management as it determines disease prognosis, morbidity, and mortality (Arrigo et al., 2020). Bioelectrical impedance analysis (BIA) and bioelectrical impedance vector analysis (BIVA) is a non-invasive, affordable, quick, and tested method to accurately assess body composition and fluid status in clinical practice (Marra et al., 2019).

### Bioelectrical Impedance

BIA uses bioelectrical impedance, described as resistance to flow of alternating current (Khalil et al., 2014). Bioimpedance is a composite measure that includes resistance and reactance. Biologically, electrical resistance is inversely related to total body water (TBW; Di Somma et al., 2014b) and therefore as the TBW increases, such as in edema, resistance decreases. Conversely, reactance is primarily related to capacitance of the cell membrane, thus reporting body cell mass (Kyle et al., 2004a; Walter-Kroker et al., 2011). Therefore, an increase in total cell body mass results in an increase in reactance. Clinically, these measures are used to derive some useful body composition parameters including intracellular body water (ICW), extracellular body water (ECW), body fat mass, and fat free mass.

Based on frequency there are 2 types of BIA: the initial single 50 kHz frequency BIA (SF-BIA), and the more recent multiple frequency BIA (MF-BIA), 1 kHz–500 kHz (Kyle et al., 2004a). Using separate frequencies is beneficial as it allows for ECW and ICW assessment, because high frequencies allow penetration of cell membrane and assessment of ICW whereas low frequencies are not able to penetrate cell membranes (Marra et al., 2019) and therefore provide assessment of TBW. These assessments enable calculation of fat free mass (Haverkort et al., 2015), together with giving an estimation of interstitial fluid or oedema (Marra et al., 2019). Although using BIA has benefits in body composition assessment, there are limitations regarding the equation used to calculate these compositions, as the measurement is influenced by factors such as body shape abnormalities, races, extreme body mass index (Kyle et al., 2004a), and fluid imbalance (Haverkort et al., 2015).

**Figure 1.**
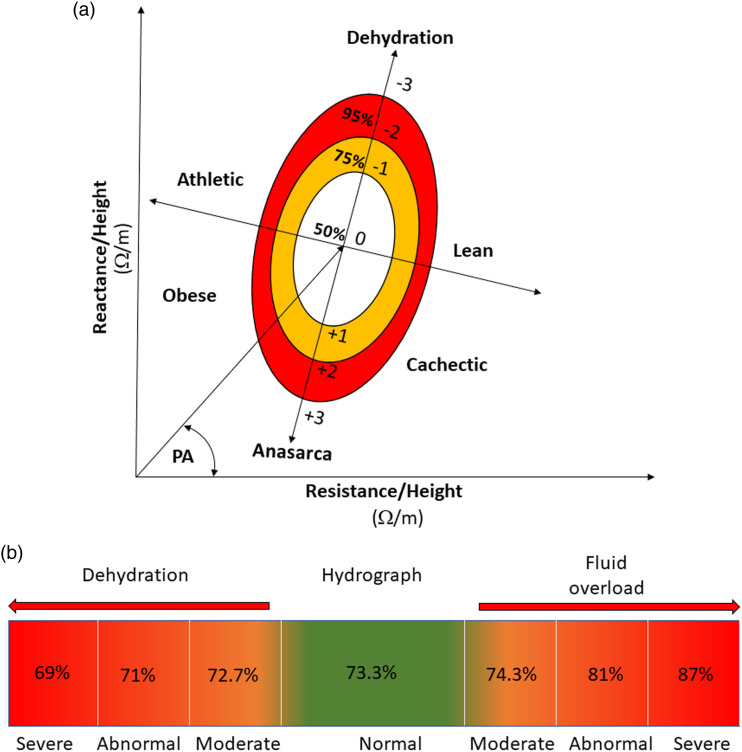
(A) BIVA ellipse indicates (1) volume overload in chronic heart failure is a bivariate vector falls outside 50% ellipse (yellow), and in acute heart failure is a bivariate vector falls outside 75% ellipse (red); (2) cachexia is identified when the bivariate vector falls outside 95% ellipse at right lower quadrant. PA = arctan(reactance/resistance) × (180°/π). This PA is drawn to be 45° for illustrative purposes. (B) Hydrograph indicates fluid status; fluid overload is when hydration index is over 74.3%.

A derivative of BIA, BIVA has been used to assess nutritional and fluid status by plotting a bivariate vector analysis of reactance and resistance standardised by height and overcome the limitations of BIA (Norman et al., 2012) (Figure 1A). Unlike BIA, BIVA does not rely on a regression equation or body weight to assess body composition as it uses raw impedance measurements (Castizo-Olier et al., 2018), thus, it can be used under diverse alterations of weight and fluid volume (Nwosu et al., 2019). To help understand the parameters measured by these techniques, some definitions are provided in Table 1.

**Table 1.** BIA Parameters, Description and Normal Values.

Parameters	Description and normal values
Edema index	The ratio of ECW to TBW are obtained from BIA (Lyons et al., 2017) An edema index >0.39 is clinically recognised as the threshold for fluid overload (Lyons et al., 2017)
BIVA	An ellipse nomograph (Figure 1A) provides estimates of body composition (*X* axis) and fluid status (*Y* axis). The ellipse is generated by using means of bivariate vector of resistance and reactance standardised by height from a healthy population to calculate 95% and 75% confidence intervals (Figure 1A) (Piccoli et al., 1994)
Phase angle	An arc tangent of reactance to resistance (Figure 1A) correlates to cellular characteristics, such as membrane integrity, cell mass and hydration (Stapel et al., 2018) and provides a composite measure for cellular health.
Hydration index	A measure reports the degree of hydration: (Dehydration, normal hydration and hyper hydration) and is generated from BIVA (Valle et al., 2011) (Figure 1B). A normal hydration index is 72.7–74.3% which is equal to 50% on the BIVA ellipse (Figure 1A) (Di Somma et al., 2014b)
Fluid distribution	An impedance ratio measures at low (5 kHz) and high (200 kHz) frequencies (200/5 kHz), and this ratio might be utilised to demonstrate ICW and ECW compartments, respectively. Abnormal fluid distribution can be indicated when the value of 200/5 kHz is ≥0.85 (Castillo-Martínez et al., 2020)

## Literature Review

A literature review was conducted to examine how BIA and BIVA are used in heart failure patients and whether it is useful in heart failure treatment and management. A systematic search was conducted to identify relevant studies related to the topic area via MEDLINE from 2002 to 19 April 2022 using search terms; ‘electric impedance’, ‘bioelectrical impedance vector analysis’, ‘BIVA’, ‘heart failure’. Inclusion and exclusion criteria to select papers were applied. Inclusion criteria were research paper in which the main aim of study is to examine whether using BIA and BIVA can benefit heart failure patients in the hospital setting. Exclusion criteria were studies using other types of bioimpedance as the main aim of the paper and studies using BIA to investigate the effect of a specific drug, non-human study.

**Figure 2.**
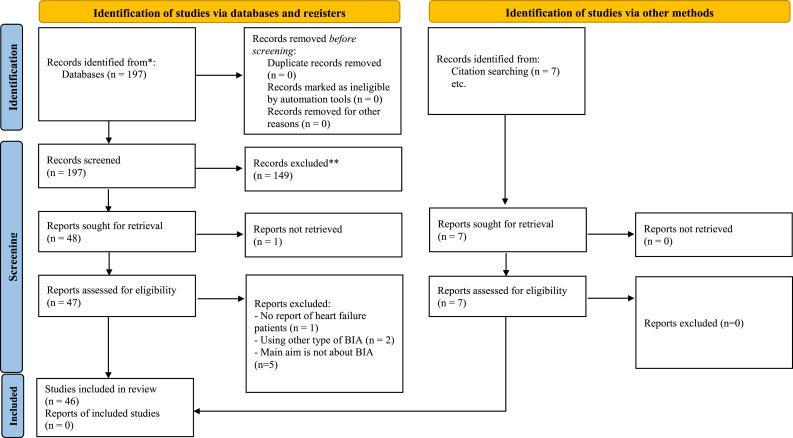
PRISMA flow diagram.

One-hundred and ninety-seven studies were identified through the database, a shown in Figure 2 (Page et al., 2021). Seven papers were identified using citation searching. Therefore, two-hundred and four papers were screened in total. The papers were selected according to the inclusion and exclusion criteria, and therefore, 48 papers were fully reviewed, and 46 papers were included for analysis. The main themes of the uses of BIA and BIVA parameters are to facilitate diagnosis of heart failure, heart failure fluid assessment and management, predict prognosis, and assess nutritional status. The parameters used in each main theme are summarised in this review together with their limitations and contraindications, as well as implications for using these measures in clinical practice.

### Diagnosis Heart Failure, Fluid Assessment and Management

BIA and BIVA have been used in acute and chronic heart failure to assess fluid status to diagnose and manage heart failure (Supplement 1).

### Diagnosis of Heart Failure

Biological markers, B-type natriuretic peptide (BNP) and N-terminal pro-BNP (NT-proBNP), are used to identify the degree of heart failure (Ponikowski et al., 2016). However, accuracy of these markers can be questioned as levels can be affected by multiple factors, such as kidney disease (Gil Martínez et al., 2016), liver dysfunction, and anemia (McDonagh et al., 2021). As BIA can assess fluid status, it has a potential to diagnose heart failure. BIA assessed fluid status has been examined and compared against BNP and NT-proBNP to accurately diagnose heart failure using area under the curve (AUC) derived from receiver operating characteristic analysis (Génot et al., 2015; Gil Martínez et al., 2016). Using reactance alone to diagnose acute heart failure (AUC = 0.76) was inferior to BNP (AUC = 0.92) (Génot et al., 2015), while using resistance/height (AUC = 0.83, 95% confidence interval (CI) 0.75–0.92), and reactance/height (AUC = 0.80, 95% CI: 0.70–0.89) to diagnose acute heart failure was as good as using ultrasound of maximum and minimum inferior vena cava (AUC = 0.90, 95% CI: 0.84–0.96 and AUC = 0.93, 95% CI: 0.87–0.98) and NT-proBNP (AUC = 0.84, 95% CI: 0.74–0.93) (Gil Martínez et al., 2016). Indeed, in multiple logistic regression analysis, the combination of BIA and BNP levels can be a strong predictor for the presence of acute decompensated heart failure (odds ratio: 40.1408, 95% CI: 5.0456 to 319.3434, *p* = 0.0005; Parrinello et al., 2008).

Interestingly, the edema index (the ratio of ECW to TBW) showed the highest sensitivity and specificity of 78% and 96%, respectively, compared to orthopnea (sensitivity and specificity: 28% and 90%), pretibial edema (72% and 92%), pulmonary congestion (68% and 86%), and rales (42% and 90%) in detecting fluid congestion to help diagnose acute heart failure (Park et al., 2018). Also, a moderate correlation between high edema index at lower extremities and log BNP was reported (*r* = 0.603, *p* < 0.001) (Park et al., 2018).

These data suggest that BIA and BNP, NT-proBNP levels, provide similar accuracy of diagnosis, and they may be used interchangeably; however, the limitation of BIA when used in patients with unstable fluid status may lead to inaccurate results. This issue with BIA can be solved by using BIVA. The BIVA generated hydration index can improve diagnosis of acute heart failure when BNP levels are undecisive (100–400 pg/mL) and using BNP levels in conjunction with the hydration index, the diagnostic ability (AUC) in this regard was reported as 0.77 with 65.3% sensitivity and 78.8% specificity (*p* < 0.0001). This combination increased the net diagnosis of acute heart failure increased from 19% (*p* = 0.016) to 77% (*p* < 0.001) (Di Somma et al., 2014a). This suggests that BIVA in combination with BNP is superior to BIA in diagnosing heart failure.

### Fluid Assessment and Management

Fluid overload in acute heart failure produces a bivariate vector that fell outside of 75% ellipse (Alves et al., 2015; Kammar-García et al., 2021; Massari et al., 2016) with 75% sensitivity and 86% specificity, as shown in Figure 1A. While in chronic heart failure, the cut-off point was a bivariate vector that falls outside of 50% ellipse with 85% sensitivity and 87% specificity (Massari et al., 2016). It has been suggested that the combination of BIVA and BNP levels increases the ability to detect fluid overload in heart failure, improving treatment and prevent further complications (Di Somma et al., 2010, 2014a; Santarelli, Russo, Lalle, De Berardinis, Navarin et al., 2017a), such as worsening renal function (Valle et al., 2011).

A number of bioimpedance-related measures including edema index, ECW, PA, resistance, reactance, and hydration index have been used to identify fluid status and degree of fluid congestion. The edema index has been used to guide fluid removal in acute heart failure patients (Yamazoe et al., 2015) by directly guiding diuretic therapy as a 0.01 increase in normal edema index equates to a 1 Kg increase edematous fluid which needs to be removed (Yamazoe et al., 2015). Edema index was also used to define cardiorespiratory fitness and functional capacity. It was reported to be inversely related to peak VO_2_ (rho = −0.307, *p* = 0.009) and exercise time (rho = −0.314, *p* = 0.006) (Marawan et al., 2021). This may be explained because then edema index identified increased ECW causing lung congestion and consequent decreased exercise capacity. Also, there was a strong correlation between weight loss and ECW (*r* = 0.766, *p* < 0.001) (Sakaguchi et al., 2015). However, there are limitations of BIA that parameters generated can be affected by the fluctuation of fluid and differences in equations used, and therefore, using BIVA might be more useful to assess fluid status in heart failure patients as reported in the evidence.

PA is inversely related to fluid overload (Table 1). There is a weak negative correlation between ECW and PA (*r* = −0.367, *p* ≤ 0.0001) (Colin-Ramirez et al., 2006). PA was significantly lower in NYHA class III-IV than I-II in both systolic (*p* = 0.04) and diastolic heart failure (*p* = 0.01) (Castillo Martinez et al., 2007). The decreased PA, therefore, significantly was related to fluid overload (*p* < 0.05) (Colin-Ramirez et al., 2006) and high risk of acute decompensation (Gulatava et al., 2021). PA also gradually reflects a decrease in fluid volume. This has been seen with fluid loss following intensive diuretic therapy, where the mean PA increased from 3.61 ± 0.82 (hospitalisation) to 3.83 ± 0.74 (on discharge) (mean ± standard deviation (SD)), and the 95% CI of this change was reported 0.15, 0.29; (De Ieso et al., 2021). PA can potentially identify nutritional status as well as hydration level (Gulatava et al., 2021); however, PA may be a better tool to assess fluid status (Scicchitano et al., 2020).

Furthermore, when hydration index is over 74.3% (50% BIVA ellipse) (Di Somma et al., 2014b) (Figure 1B), this indicates hyper-hydration (Di Somma et al., 2014a; Génot et al., 2015; Valle et al., 2011). This hydration index can be used to guide diuretic treatment as the index decreases rapidly following fluid removal from admission to discharge (76.74 ± 4.0 vs. 74.4 ± 2.0 (*p* < 0.0001; Di Somma et al., 2010) and (82.8 ± 6 vs. 78.5 ± 6 (*p* < 0.001; mean ± SD; Santarelli, Russo, Lalle, De Berardinis, Vetrone et al., 2017b). This demonstrates its potential to monitor treatment effect, together with being a diagnostics tool. In summary, although BIA can be used to facilitate heart failure treatments and managements, BIVA and its derived measures seem to be more accurate values to manage and monitor heart failure than BIA.

### Using BIA and BIVA for Predicting Prognosis

Parameters calculated using BIA and BIVA, such as body compositions, edema index, hydration status, and PA can predict prognosis (Supplement 2). In chronic heart failure patients, those with a high lean body mass and body fat mass index had better 5-year clinical outcomes and better survival rates than those with low lean body mass (89.3% vs. 80.9%, *p* = 0.036) and body fat mass index (90.2% vs. 80.1%, *p* = 0.008) (Thomas et al., 2019). This phenomenon is known as the obesity paradox, which states that heart failure patients with obesity had better prognoses than heart failure patients who were normal weight and underweight, regardless of their ejection fraction status (heart failure with preserved ejection fraction, heart failure with reduced ejection fraction) (Carbone et al., 2019).

An increased edema index is associated with increased rates of all-cause mortality, urgent transplant, or insertion of ventricular assistant device (Lyons et al., 2017). Using the edema index combined with a multidisciplinary approach in acute heart failure patients can reduce rehospitalisation (3.8%) compared to a control group (18.9%) or a case management group (13.2%, *p* = 0.03; Liu et al., 2012). Moreover, abnormal fluid distribution together with low grip strength in men was independently related to all-cause mortality (hazard ratio 2.8; 95% CI: 1.25–6.4; *p* = 0.01), and this combination of parameters could suggest advanced heart failure regardless of gender (Castillo-Martínez et al., 2020). However, using BIA to examine prognostic values remain controversial. As Curbelo et al. (2019) reported, BIA parameters did not show prognostic values (Curbelo et al., 2019), and this might be due to the use of SF-BIA rather than MF-BIA and BIVA that probably affects the results due to the equation and its ability to penetrate cells. This, therefore, introduces the use of BIVA.

BIVA can also help predict cardiovascular events after discharge. Using a threshold hydration index level of 74.3%, acute heart failure patients with a higher hydration index had higher deaths and rehospitalisation rates than patients with a lower hydration index level (83.7 ± 7% vs. 80 ± 7%, *p* < 0.008) (Di Somma et al., 2014a) and (82.2 ± 4.8 vs. 73.7 ± 2.0, *p* < 0.0001, mean ± SD; Villacorta et al., 2021). The mortality and readmission rates were higher in patients with hyper-hydration index (>74.3%) than patients with normal hydration index (<74.3%, and >72.7%, Figure 1B) (3.28 and 3.83 per 10 persons-years vs. 1.43 and 2.68 per 10 persons-years, (*p* < 0.05; Núñez et al., 2016). Also, acute heart failure patients with the severe hyperhydration, hydration index 87.1%–100%, had a longer length of stay in the hospital than those with normal hydration (9.04 days [IQR: 8.85–9.19 d] vs. 7.36 days [IQR: 7.34–7.39 d], *p* < 0.05; Massari et al., 2019). Furthermore, use of a combination of using BIVA parameters, BNP, hydration index, estimated plasma volume status, and BUN/creatinine ratio together, is a useful predictor of mortality risk (Massari et al., 2020).

PA was adversely associated to mortality rates as a PA was significantly lower in non-survivor group than survival group in acute heart failure (4.3 [IQR: 3.4–5.6] vs. 3 [IQR: 2.1–3.9], *p* < 0.0001 (Kammar-García et al., 2021); 6.3 ± 2.2 versus 5.08 ± 1.9, mean ± SD, *p* < 0.038 (Alves et al., 2016)). Additionally, the relative risk (RR) for the association with all-cause mortality in a group with lowest PA < 4.2 was reported (RR = 3.08, 95% CI: 1.06–8.99) compared to the group with highest PA ≥ 5.7 (RR = 1) (Colín-Ramírez et al., 2012). Therefore, low PA can be used as a prognostic marker. Moreover, PA was used with galectin-3 levels, a biomarker representing cardiac fibrosis, to predict prognosis. A reduced PA and elevated galectin-3 levels significantly relates to hospitalisation at 60 days (AUC = 0.625, *p* = 0.003), 180 days (AUC = 0.545, *p* = 0.05) and 18 months (AUC = 0.620, *p* = 0.04) and mortality at all time points (1, 2, 3, 6, 12, 18 months) (*p* < 0.005) (De Berardinis et al., 2014). The benefit of using this combination of biomarkers help describe both degree of cardiac fibrosis/remodelling and fluid status. Therefore, parameters derived from BIVA -hydration index and PA-seems to be better prognostic markers than BIA.

### Nutritional Assessment Using Bioelectric Impedance Measures in Heart Failure Patients

BIAs and BIVA have important roles in measuring body compositions among heart failure patients and identifying their nutritional status (Supplement 3). BIA frequency is important when assessing body compositions and there are 2 types of BIAs: SF-BIA and MF-BIA. MF-BIA has been used in heart failure patients due to the fact that multiple frequencies provides more accurate assessment of body water and therefore body cell mass, which improves accuracy of consequent anthropometric measurements (Liu et al., 2012). Hence, MF-BIA has also been used to assess body composition to identify malnutritional status; sarcopenia (Ogawa et al., 2020), and cardiac cachexia in heart failure patients (Castillo-Martínez et al., 2012; González-Islas et al., 2020; Hirose et al., 2020). There was a significantly negative correlation between parameters generated by MF-BIA -PA and reactance- and C-reactive protein level -inflammatory marker used to diagnose cachexia (*p* < 0.01). This might relate to the occurrence of cachexia (Sobieszek et al., 2019).

Compared to SF-BIA, MF-BIA accuracy was proven to be as good as dual-energy X-ray absorptiometry (DEXA) with no differences in mean (mean (standard deviation) of DEXA versus MF-BIA: body fat 28(6) versus 27(9); fat mass 20(6) versus 20(9); fat free mass 52(10) versus 53(11) (Alves et al., 2014). There were also strong correlations between determination of lean mass (*r* = 0.95), fat mass (*r* = 0.96) and body mass (*r* = 0.84) between MF-BIA and DEXA (Shah et al., 2021). However, it is suggested not to use them interchangeably due to mean differences of fat mass (mean difference −5.1 kg) and lean mass (mean difference 5.5 kg) in both methods (Shah et al., 2021). Thus, although MF-BIA is more accurate than SF-BIA and reported high correlation with DEXA, there was a wide limit of agreements for MF-BIA reported, which was believed to be due to a nonlinear distribution, leading to a need for an appropriate regression equation (Alves et al., 2014). Due to this reason and the limitations of BIA as previously mentioned, vectorial analysis of the BIA parameters (BIVA) should be considered and used in heart failure management.

BIVA has been utilised in heart failure patients to assess nutritional status (Figure 1A). A decreased PA suggests nutritional status anomalies, such as cachexia, sarcopenia and malnutrition in chronic heart failure patients (Castillo-Martínez et al., 2012; González-Islas et al., 2020; Hirose et al., 2020). A positive correlation between PA and body mass index was reported for males and females *r* = 0.3310 (*p* < 0.0001) and *r* = 0.3115 (*p* < 0.001), respectively (Hirose et al., 2020).

In conclusion, according to the evidence, BIVA seems to be more accurate to identify nutritional status in heart failure patients than BIAs due to the conditions of heart failure, such as abnormal fluid status, and inconsistency of findings when comparing BIA to DEXA that might result in inaccurate results. Although BIA and BIVA benefit heart failure assessment and management, safety concerns regarding using BIA and BIVA have been reported and examined to ensure their safety, such as interference of BIA to pacemaker’s function. The issues will be explored below.

### Contra-Indications of BIA in Heart failure Patients

Despite the potential advantages of BIA, there are some notable contra-indications in heart failure patients associated with cardiac implantable electronic devices (Cornier et al., 2011; NIHR, 2016). A potential for BIA to interfere with electrical current of pacemakers and defibrillators resulting in malfunction of the device, signal oversensing or stimulation inhibition, has been reported (Fabregat-Andrés et al., 2015; Kyle et al., 2004b). However, more recent studies tested the safety of BIA in heart failure patients and reported no interference with battery and functions of cardiac implantable electronic devices (CIED) and cardiac resynchronization therapy (Buch et al., 2012; Chabin et al., 2019; Fabregat-Andrés et al., 2015; Garlini et al., 2020; Meyer et al., 2017; Roehrich et al., 2020) (Supplement 4). Following this, some versions of BIA were shown to be safe to use, under manufacturer guidance.

### Implication for Practice

BIVA is more advantageous in heart failure screening, treatments, and management, including determining fluid and nutritional status than BIAs. BIVA and hydrograph have potential benefits as they can be used to identify chronic or acute heart failure, facilitate heart failure treatment by avoiding complications when adjusting diuretic treatment in acute settings and monitoring fluid status. Currently, the American Heart Association recommends BIVA to optimise fluid treatment to avoid cardiorenal syndrome (Rangaswami et al., 2019). The combination of using BIVA and serum BNP or NT-proBNP levels also increase capabilities to guide heart failure treatments and predict prognosis in heart failure patients.

Despite the benefits of bioimpedance measurements, safety concerns must be acknowledged. In cased where BIA measurement cannot be performed due to concerns regarding contraindications, an alternative method to measure anthropometry or assessing fluid status should be considered. In addition to safety concerns, it is worth noting that although the statistical significances were reported in the findings of this review, the effect size of some included studies might be small and therefore should be interpreted with caution considering clinical applicability. Furthermore, due to lack of consistency in reports of BIA/BIVA parameters leading to difficulties in combining analyses, future studies should report BIA/BIVA parameters, such as PA, edema index, hydration index, reactance/height and resistance/height, if applicable as these parameters tend to be accurate measurements that would further benefit heart failure management and research, particularly, in a systematic review and meta-analysis to further investigate on which parameters would comprehensively reflect conditions of heart failure patients.

## Conclusion

This review has demonstrated the uses of BIA and BIVA in acute and chronic heart failure patients. It also emphasises the importance of using BIA and BIVA to screen and detect for malnutrition, assess, and monitor fluid status to provide treatment, predict prognosis, including the safety concern of using BIA. Indeed, BIVA and its parameters, such as PA and hydration index, seem to be more superior than BIA in heart failure patients. The combinations of using BIVA and BNP/NT-proBNP increases the ability to detect heart failure and predict prognosis. However, further studies are required to examine the replacement of current practice by using BIVA, including cost effectiveness. Further work is needed on determining the effects of BIA on patients with CIED.

## Supplemental Material

Supplemental Material - A Current Review of the Uses of Bioelectrical Impedance Analysis and Bioelectrical Impedance Vector Analysis in Acute and Chronic Heart Failure Patients: An Under-valued Resource? Click here for additional data file. Supplemental Material for A Current Review of the Uses of Bioelectrical Impedance Analysis and Bioelectrical Impedance Vector Analysis in Acute and Chronic Heart Failure Patients: An Under-valued Resource? by Jenjiratchaya Thanapholsart, Ehsan Khan and Geraldine A. Lee in Biological Research For Nursing
