# Supplementary material for: A Current Review of the Uses of Bioelectrical Impedance Analysis and Bioelectrical Impedance Vector Analysis in Acute and Chronic Heart Failure Patients: An Under-valued Resource?
Source: Biol Res Nurs. 2022 Nov 7;25(2):240–9. doi: 10.1177/10998004221132838 (PMC10021121; doi:10.1177/10998004221132838)
Supplement: Supplemental Material - A Current Review of the Uses of Bioelectrical Impedance Analysis and Bioelectrical Impedance Vector Analysis in Acute and Chronic Heart Failure Patients: An Under-valued Resource? [file sj-pdf-1-brn-10.1177_10998004221132838.pdf]

Supplementary information

Supplement1: the uses of BIA and BIVA to assess fluid status indicating tools, aims and results of study, recruiting patients with cardiac devices

Author	Population	Tools<br>(type of BIA; country made; BIA frequency)	Aims of study relate to the use of BIA or BIVA	Results	Include patients with CIED
Colin-Ramirez et al. (2006)[1]	Chronic heart failure patients (n=132)	-MF-BIA (BodyStat QuadScan 4000; Bodystat; 5, 50, 100, and 200 kHz)	- To assess volume overload and anaemia in heart failure	Systolic HF (EF<45%) with anaemia had lower BMI, weight, waist and hip circumferences and PA, less grip strength and higher ECW than those with non-anaemic patients in systolic and diastolic HF.	N/A
Castillo Martinez et al. (2007) [2]	Chronic heart failure patients (n=243)	-MF-BIA (BodyStat QuadScan 4000; Bodystat; 5, 50, 100, and 200 kHz)	- To examine the usefulness of BIVA parameters in HF with systolic dysfunction and preserved systolic function according to NYHA class.	Patients with HF with NYHA III-IV class were reported more fluid accumulation, lower PA, and more downslope and shorter vector in the BIVA ellipse than NYHA I-II.	N/A
Parrinello et al. (2008) [3]	Acute heart failure patients (N=292, n=172)	- SF-BIA (BIA-101, Akern Srl, Florence, Italy; 50 kHz)	To assess and compare the uses of BIA to other parameters; BNP, to differentiate ADHF and other conditions causing dyspnoea.	- ADHF vs non-ADHF had 100% dyspnoea, but the degree of congestion identified by BIVA and BNP helped identifying ADHF. BNP levels were reported higher in ADHF than non-ADHF and control group (591.8±501 pg/ml. vs 69.5±42 pg/ml vs 47.6±20 pg/ml, p<0.001) (mean±SD) whereas the whole BIA parameters for Rz and Xc in these 3 groups were reported 402.3 ± 55.5 and 31 ± 7.5 Ohm vs. 513.2 ± 41.8 and 49.2 ± 7 ohms vs. 547 ± 32.8 and 54 ± 5.2 ohms (p<0.0001), respectively.<br>- The Rz and Xc significantly increased in both whole body and segmental at admission and discharge (p<0.0001) whereas among non-ADHF showed non-significance.<br>- The AUC of using BIA + BNP levels to differentiate dyspnoea caused by ADHF and by others was reported highest (AUC=0.989) compared to other parameters.<br>- Multiple logistic regression analysis, the strong predictor of ADHF was the combination of BIA and BNP levels.	N/A
ADHF= acute decompensated heart failure, AUC=area under curve, BIVA= bioelectric impedance vector analysis, BIA= bioelectric impedance analysis, BNP= B-type natriuretic peptide, CIED= cardiovascular implantable electronic devices, ECW= extracellular water, MF-BIA= multiple frequency BIA, N/A = data not available, n=heart failure patients, NYHA= New York heart association, PA= phase angle, SD= standard deviation, SF-BIA= single frequency BIA					

Supplementary information

Supplement1: the uses of BIA and BIVA to assess fluid status indicating tools, aims and results of study, recruiting patients with cardiac devices

**Author**	**Population**	**Tools** (type of BIA; country made; BIA frequency)	**Aims of study relate to the use of BIA or BIVA**	**Results**	**Include patients with CIED**
Somma et al. (2010)[4]	Acute heart failure patients (n=51)	N/A	- To test the validity of BIVA for assessing fluid congestion, including correlation between BIVA and BNP levels and their ability to guide heart failure management.	- Hydration index at admission, 24 and 72 hours after admission and before discharge substantially decreased after diuretic treatments (p<0.001).<br>- Hydration index > 80.5% was associated with mortality or rehospitalization for cardiogenic shock at 90 days (p<0.04).<br>- Using hydration index together with BNP levels are recommended to guide treatment and management in heart failure.	N/A
Valle et al. (2011)[5]	Acute heart failure patients (n=300)	- A tetrapolar electrical impedance analyser and SF-BIA (Cardio EFG software; Akern, Pontassieve, Florence, Italy; 50kHz)	- Using BIVA and BNP to reduce congestion to optimise LoS and decrease long-term complications<br>- Using BIVA can decrease the occurrences of worsening of renal function.	- Using BIVA to assess hydration index and BNP guided management reduced events rates of death and hospitalisation after discharge and the occurrence of worsening of renal function due to avoiding unnecessary diuretic prescription.<br>- The group that responded rapidly to treatments compared to late- or non- responses had significantly shorter LoS.	N/A
Somma et al. (2014)[6]	Acute heart failure patients (N=381, n=270)	- A tetrapolar electrical impedance analyser and SF-BIA (Cardio EFG software; Akern, Pontassieve, Florence, Italy; 50kHz)	- To identify the accuracy of hydration index and using hydration index to examine prognosis after 1-month discharge.	- The hydration index shows significantly higher in acute heart failure groups than control (no heart failure).<br>- In patients that have BNP in the grey zone, hydration index > 79.2% shows a significant diagnostic power (sensitivity of 65.3% and specificity of 78.8%) (p<0.0001).<br>- Hydration index > 73.4% did not show significant results in detecting acute heart failure (p<0.08) compared to BNP > 141.5 pg/ml.<br>- Patients who had higher value of hydration index in acute heart failure group had higher readmission and death rates than others.	N/A
Alves et al. (2015)[7]	Acute heart failure patients (n=57)	- SF-BIA (Biodynamics Corp., Seattle, Washington, USA; 50kHz)	- To assess BIVA and PA whether it reflect fluid retention at admission, discharge and 90 days after discharge	- BIVA parameters Rz/H and Xc/H were significantly changed with the reduction of fluid retention from the admission day to discharge day, but there was no change from home discharge to 90 days after discharge.	No

BIVA= bioelectric impedance vector analysis, BIA= bioelectric impedance analysis, BNP= B-type natriuretic peptide, CIED= cardiovascular implantable electronic devices, LoS = length of stay, MF-BIA= multiple frequency BIA, N/A = data not available, n=heart failure patients, PA= phase angle, SF-BIA= single frequency BIA, Rz/H =resistance/height, Xc/H=reactance/height

Supplementary information

Supplement1: the uses of BIA and BIVA to assess fluid status indicating tools, aims and results of study, recruiting patients with cardiac devices

Author	Population	Tools<br>(type of BIA; country made; BIA frequency)	Aims of study relate to the use of BIA or BIVA	Results	Include patients with CIED
Génot et al. (2015)[8]	Acute heart failure patients (N=77, n=37)	- Z-Metrix BioparhΩm device	- The main aim was to assess the diagnostic performance of BIVA and compare it against BNP and echocardiographic parameters for diagnosing acute heart failure with dyspnoea	- Reactance value can identify the differences between patients with acute heart failure and without while other value generated by BIA cannot.<br>- Reactance was reported a sensitivity of 67% sensitivity and 76% specificity with area under curve = 0.76 while AUC of BNP levels was reported 0.92.	N/A
Sakaguchi et al. (2015)[9]	Acute heart failure patients (n=130)	- MF-BIA (BioScan 920-2 analyzer, Maltron International, Rayleigh, Essex, UK; 5, 50, 100, 200 kHz)	- To create and test a method to identify the amount of fluid accumulation using MF-BIA to assist AHF management	- The regression equations were created to predict ECW for Japanese, however, the level of ECW predicted by the created equations (P) were significantly lower than the ECW measured by MF-BIA (M). The authors then calculated the M/P ratio of ECW. This ratio was dynamically changed by fluid removal.<br>- This M/P ratio of ECW can also be used to predict prognosis. When M/P ratio of ECW was higher than one, the occurrences of cardiac death and rehospitalisation were higher than M/P ratio of ECW below 1 (HR 5.28 (95% CI 2.21-12.6), p<0.0001).<br>- There were 37 cardiac events in 6 months after home discharge; 35 rehospitalisation and 2 sudden cardiac deaths.<br>- The amount of ECW on admission and discharge were significantly different (p<0.001).<br>- Body weight loss and ECW reduction were strongly and significantly correlated (r=0.766, p < 0.001)	N/A
Yamazoe et al. (2015) [10]	Acute heart failure patients (n=60)	- MF-BIA (InBody S10; Biospace device, Soul, Korea; 1, 5, 50, 250, 500, and 1000 kHz)	- To identify if BIA could anticipate the dry weight in removing clinical congestion at the time of admission.	- When there was an increase of 0.01 of normal oedema index, a 1 kilogram reduced in body weight was needed	No
BIVA= bioelectric impedance vector analysis, BIA= bioelectric impedance analysis, BNP= B-type natriuretic peptide, CIED= cardiovascular implantable electronic devices, ECW = extracellular water, HR=hazard ratio, MF-BIA= multiple frequency BIA, N/A = data not available, N= total population, n=heart failure patients, PA= phase angle, SF-BIA= single frequency BIA, Rz/H =resistance/height, Xc/H=reactance/height					

Supplementary information

Supplement1: the uses of BIA and BIVA to assess fluid status indicating tools, aims and results of study, recruiting patients with cardiac devices

**Author**	**Population**	**Tools** (type of BIA; country made; BIA frequency)	**Aims of study relate to the use of BIA or BIVA**	**Results**	**Include patients with CIED**
Martínez et al. (2016)[11]	Acute heart failure patients (N=96, n=57)	- SF-BIA (Cardio EFG; Akern, Florence, Italy; 50kHz)	To compare the accuracy of ultrasound inferior vena cava, BIA and NT-proBNP for diagnosing acute heart failure, including pair cut-off values with recent evidence.	- Using ultrasound inferior vena cava, BIA and NT-proBNP to diagnose acute heart failure by assessing volume shows no differences between them.<br>- In patients with kidney disease, using NT-proBNP had significantly contrast with eGFR (p<0.0007), therefore, in this patients BIA or ultrasound are recommended to make a diagnosis of acute heart failure patients rather than NT-proBNP.<br>- Area under the curve of NT-proBNP, Rz/H, Xc/H were 0.84, 0.83, 0.80 respectively for diagnosis acute heart failure and no significant different among these three.	N/A
Massari et al. (2016) [12]	Acute and chronic heart failure patients (n=900)	- SF-BIA (Cardio EFG; Akern, Florence, Italy; 50kHz)	- Compare between BIVA and BNP to detect oedema	- ADHF higher BNP and congestion and lower BIVA parameters than CHF.<br>- High level BNP alone could not be related to fluid overload.<br>- BIVA can detect fluid overload better than BNP.<br>- The BIVA ellipse in ADHF and CHF for detection fluid congestion were the lower pole of the 75th and 50th percentile, respectively.	Yes
Santarelli et al. (2017) [13]	Acute heart failure patients (n=292)	- SF-BIA (Cardio EFG; Akern, Florence, Italy; 50kHz)	- Using BIVA to measure the decreased congestion during hospitalisation and predict 90 days events	- BIVA (Rz/H and Xc/H) at D0 and discharge were reported significantly different.<br>- BIVA can be used to detect peripheral fluid overload in AHF as the vector show wet BIVA while patients without AHF had no vector reported.<br>- BIVA + clinical signs (ie. rale) at discharge can be substantially predicted for cardiovascular events events 90 days after discharge AUC 0.97, p <0.0001	N/A
Santarelli et al. (2017)[14]	Acute heart failure patients (N=336, n=221)	- SF-BIA (Cardio EFG; Akern, Florence, Italy; 50kHz)	- To examine the ability to detect changes of degree of fluid congestion from hospitalisation day to discharge day by BIVA.	- Hydration index at hospital admission was significantly higher in acute heart failure patients than no acute heart failure patients (p<0.001).<br>- Hydration index at discharge significantly reduced among acute heart failure patients (p<0.001).	N/A
ADHF = acute decompensated heart failure, AUC = area under curve, BIVA= bioelectric impedance vector analysis, BIA= bioelectric impedance analysis, CHF= chronic heart failure, CIED= cardiovascular implantable electronic devices, MF-BIA= multiple frequency BIA, N/A = data not available, N= total population, n=heart failure patients, NT-proBNP= N-terminal pro-B-type natriuretic peptide, NYHA= New York heart association, PA= phase angle, SF-BIA= single frequency BIA, Rz/H =resistance/height, Xc/H=reactance/height					

Supplementary information

Supplement1: the uses of BIA and BIVA to assess fluid status indicating tools, aims and results of study, recruiting patients with cardiac devices

Author	Population	Tools (type of BIA; country made; BIA frequency)	Aims of study relate to the use of BIA or BIVA	Results	Include patients with CIED
De Ieso et al. (2021) [15]	Acute heart failure patients (n=142)	- MF-BIA (seca® mBCA 515, Hamburg, Germany)	To investigate whether the changes of hydration status derived from BIVA can be used to monitor intensified diuretic therapy.	- There was a significant association of the daily changes of TBW and ECW with body weights.<br>- The most accurate parameter to monitor rapid changes in hydration status was ECW during intensified diuretic therapy.<br>- The mean±SD of PA elevated from baseline 3.61 ± 0.82° at hospitalisation to 3.83 ± 0.74° at the last measurements (95% confidence interval of this change is [0.15, 0.29]).<br>- There was a negative association between weight/fluid loss and Rz and Xc.	No
Marawan et al. (2021) [16]	Chronic heart failure patients (n=72)	-SF-BIA (RJL System, Inc, Clinton Township, MI)	To examine if cardiorespiratory fitness in chronic heart failure patients with type2 diabetes mellitus can be predicted by oedema index, and to test the hypothesis that increase oedema index would anticipate decreased cardiorespiratory fitness.	-Oedema index was negatively associated with peak VO2 (rho=-0.307, p=0.009) and exercise time (rho=-0.314, p=0.006).	N/A
BIVA= bioelectric impedance vector analysis, BIA= bioelectric impedance analysis, BNP= B-type natriuretic peptide, CIED= cardiovascular implantable electronic devices, ECW= extracellular water, MF-BIA= multiple frequency BIA, N/A = data not available, N= total population, n=heart failure patients, NYHA= New York heart association, PA= phase angle, SD= standard deviation, SF-BIA= single frequency BIA, Rz =resistance, TBW = total body water, Xc =reactance					

Supplementary information

Supplement2: the uses of BIA and BIVA to predict prognosis indicating tools, aims and results of study, recruiting patients with cardiac devices

Author	Population	Tools<br>(type of BIA; country made; BIA frequency)	Aims of study relate to the use of BIA or BIVA	Results	Include patients with CIED
Colín-Ramírez et al. (2012) [17]	Chronic heart failure patients (n=389)	- SF-BIA (RJL Systems analyzer; Quantum X, Clinton Township, MI, USA; 50 kHz)	PA and parameters for prognosis.	- Low PA (PA <4.2) related to low BMI, grip strength<br>- PA related to mortality rates; the lowest survival rates reported small angles.	N/A
Liu et al. (2012) [18]	Acute heart failure patients (n=112)	- MF-BIA (Inbody 720 multifrequency analyzer; Seoul Korea, 1, 5, 50, 250, 500 and 1000 kHz)	Whether or not BIA can predict prognosis among hospitalised acute heart failure patients.	-Patients who had oedema index > 0.39 had significantly higher re-hospitalisation rate than patients with oedema index <0.39 (p<0.05).<br>- The odd ratio of univariate analysis of rehospitalisation and oedema index was 4.73 with 95% confidence interval 1.53–14.58 (p=0.01)	No
Liu et al. (2012) [19]	Acute heart failure patients (n=159)	- MF-BIA (Inbody 720 multifrequency analyzer; Seoul Korea; 1, 5, 50, 250, 500 and 1000 kHz)	To compare between using oedema index to guide heart failure management program provided by multidisciplinary, management program alone and management provided by primary care physicians.	Management plan guiding by oedema index shows substantial decreased HF- associated and all-cause-associated rehospitalisation and all events.	No
De Berardinis (2014) [20]	Acute heart failure patients (n=194)	- SF-BIA (Akern Srl, Pontassieve, Florence, Italy; 50 kHz)	To assess the prognostic prediction of GAL3, BIVA and the combination of both in short- and long- term in the emergency department	- The AUC of PA together with GAL3 significantly related to rehospitalisation at 60 days (AUC = 0.625, p = 0.003), 180 days (AUC = 0.545, p = 0.05) and 18 months (ACU = 0.620, p=0.04) whereas this combination in term of death rates was reported higher AUC with level of significance than rehospitalisation at all time points (30 days, 60days, 90 days, 180 days, 12 months, 18 months).	N/A
Alves et al. (2016)[21]	Acute heart failure patients (n=71)	- SF-BIA (Biodynamics 450: Biodynamics Corp. Seattle, Washington, USA; 50 kHz)	To assess the effect of PA as a prognostic marker on mortality rate at the hospital admission	Forty-one percent of patients died and was significantly related to low PA after 2 years follow-up period, and PA < 4.8 at the admission time was associated to death rates.	No
BIVA= bioelectric impedance vector analysis, BIA= bioelectric impedance analysis, BMI=body mass index, BNP= B-type natriuretic peptide, CIED= cardiovascular implantable electronic devices, GAL3 = galectin-3, HF= heart failure, IVC = inferior vena cava, MF-BIA= multiple frequency BIA, N/A = data not available, n=heart failure patients, NTproBNP = amino-terminal proB-type natriuretic peptide, PA= phase angle, SF-BIA= single frequency BIA, Rz/H =resistance/height, Xc/H=reactance/height					

Supplementary information

Supplement2: the uses of BIA and BIVA to predict prognosis indicating tools, aims and results of study, recruiting patients with cardiac devices

**Author**	**Population**	**Tools** (type of BIA; country made; BIA frequency)	**Aims of study relate to the use of BIA or BIVA**	**Results**	**Include patients with CIED**
Núñez et al. (2016)[22]	Acute heart failure (n=369)	- SF-BIA (bioelectrical body-hydration monitor 'CardioEFG' from Akern Srl, Italy)	To examine the rates of rehospitalisation and all-death events by using fluid status assessed BIVA	Acute heart failure patients in the hyper-hydration status group were reported to have the highest mortality and rehospitalisation rates than dehydration and euvolemia group.	N/A
Lyons et al., (2017) [23]	Chronic heart failure patients (n=359)	- MF-BIA (InBody 520 multifrequency; 5, 50, and 500 kHz)	To identify prognostic outcomes using oedema index	High oedema index showed higher rates of all-cause mortality, urgent transplant, or ventricular assist device than a normal oedema index.	N/A
Curbelo et al. (2018) [24]	Chronic heart failure (n=99)	- SF-BIA (BIA-101, Akern, Florence, Italy; 50 kHz)	To examine the benefits of using IVC ultrasonography, lung ultrasonography and BIA to predict hospitalisation and mortality due to heart failure when compared to NTproBNP.	- Parameters, such as total B-lines, IVC parameters, NTproBNP levels, except parameters generated by BIA showed significant differences between heart failure admission or death rates group and no admission or death rates ($p < 0.05$).<br>- The AUC of NTproBNP reported highest AUC (AUC=77.7, 95% confidence interval 67.5-88.0) albeit insignificance while the AUC of all BIA parameters were reported lowest.	N/A
Park et al. (2018)[25]	Acute heart failure patients (N=100, n=50)	- MF-BIA (InBody S10; Biospace device, Soul, Korea; 1, 5, 50, 250, 500, and 1000 kHz)	- Examine diagnostic implication for assessing fluid status by using BIA in patients who presented dyspnoea.	- Among patients presented pretibial oedema had significantly higher oedema index trunk, upper and lower extremities than non-acute heart failure ($p<0.0001$)<br>- High oedema index at lower extremities was moderately correlated to log BNP ($r = 0.603$, $p < 0.001$).<br>- There was the highest significant correlation between lower extremities and acute heart failure diagnosis than other segmental oedema index.	N/A

BIVA= bioelectric impedance vector analysis, BIA= bioelectric impedance analysis, BNP= B-type natriuretic peptide, CIED= cardiovascular implantable electronic devices, IVC = inferior vena cava, MF-BIA= multiple frequency BIA, N/A = data not available, n=heart failure patients, NTproBNP = amino-terminal pro-B-type natriuretic peptide, PA= phase angle, SF-BIA= single frequency BIA, Rz/H =resistance/height, Xc/H=reactance/height

Supplementary information

Supplement2: the uses of BIA and BIVA to predict prognosis indicating tools, aims and results of study, recruiting patients with cardiac devices

**Author**	**Population**	**Tools** (type of BIA; country made; BIA frequency)	**Aims of study relate to the use of BIA or BIVA**	**Results**	**Include patients with CIED**
Thomas et al, (2019)[26]	Chronic heart failure patients (n=359)	- MF-BIA (InBody 520 multifrequency; 5, 50, and 500 kHz)	- To identify the effect of body compositions on prognosis in heart failure patients	Heart failure patients with high lean body mass index and body fat mass index had better survival rates and best outcomes within 5 years follow up.	Yes
Massari et al. (2019) [27]	Acute heart failure (n=706)	- SF-BIA (Cardio EFG; Akern RJL Systems, Florence, Italy; 50kHz)	- To test the precision of BIVA when uses to predict LoS	- The increase in hydration index positively associated with increase in LoS. LoS was reported in normal hydration 7.36 days [IQR: 7.34–7.39 d] and in severe hyperhydration 9.04 days [IQR: 8.85–9.19 d] ($p<0.05$).<br>- BNP levels in severe hyperhydration were significantly high compared to normal hydration (1698±1215.03 vs 1134.02±1083.85 (mean±SD), $p<0.05$).<br>- Multivariate regression analysis using hydration index and biomarkers could predict LoS but not peripheral oedema.	Yes
Castillo-Martínez et al. (2020) [28]	Chronic heart failure (n=546)	-MF-BIA (BodyStat QuadScan 4000; Bodystat; 5, 50, 100, and 200 kHz)	-To investigate the independent relation between existing fluid alternations and decreased low hand grip strength with all-cause mortality.	- There was an independent association between the presence of abnormal fluid distribution and hand grip strength with all-cause fatality, and the hazard ratio was reported 2.8 (95% CI, 1.25–6.4; $p = 0.01$) in male only.	N/A
Massari et al. (2020)[29]	Acute and chronic heart failure patients (n=436)	- SF-BIA (Cardio EFG; Akern, Florence, Italy; 50kHz)	- To examine the effects of BIVA, BNP, estimated plasma volume status, and BUN/creatinine ratio of fluid overload on mortality	- Non-survivor had significantly higher BIVA, BNP, estimated plasma volume status ($p<0.001$), and BUN/creatinine ratio ($p=0.002$) than survivor.<br>- The combination of congestive markers together can predict 40% risks of death.	Yes
BIVA= bioelectric impedance vector analysis, BIA= bioelectric impedance analysis, BNP= B-type natriuretic peptide, LoS= length of stay, MF-BIA= multiple frequency BIA, N/A = data not available, N= total population, n=heart failure patients, PA= phase angle, Rz/H =resistance/height, SD = standard deviation, SF-BIA= single frequency BIA, TBW=total body water, Xc/H=reactance/height					

Supplementary information

Supplement2: the uses of BIA and BIVA to predict prognosis indicating tools, aims and results of study, recruiting patients with cardiac devices

Author	Population	Tools (type of BIA; country made; BIA frequency)	Aims of study relate to the use of BIA or BIVA	Results	Include patients with CIED
Kammar-García et al. (2021) [30]	Acute heart failure patients (n=142)	-MF-BIA (BodyStat QuadScan 4000; Bodystat; 5, 50, 100, and 200 kHz)	- To compare each BIA parameters to identify fluid overload and their relation to 30-day mortality in acute patients admitted to the emergency department.	- To define fluid overload, only ECW to TBW and ICW/H^2 showed no differences between these two groups while other parameters related to fluid overload, such as Z value, resistance and reactance.<br>- 94% of patients with fluid overload were identified by impedance vector analysis.<br>- Z value, resistance, reactance and PA were lower in non-survivors than survivors.<br>- Multivariate models recommend that impedance vector analysis, impedance ratio and resistance predict one-month mortality among patients admitted to the emergency department.	No
Villacorta et al. 2021 [31]	Acute heart failure (n=80)	EFG Renal software (Akern, Pontassieve, Florence, Italy)	- To examine the association of WRF and persistent congestion at discharge by using BIVA to predict long-term events; a combination of cardiac death or HF hospitalization.	- The long-term events can be predicted by hydration index > 76.5% at discharge. The number of events in patients with WRF/congestion was highest than other groups (n=9), and the hazard ratio was 1.39 [1.25-1.54] (p<0.0001).<br>- Hydration index at discharge was significantly different between a group with events (82.2±4.8) and without (73.7±2.0) (mean±SD) p<0.0001.	N/A

BIVA= bioelectric impedance vector analysis, BIA= bioelectric impedance analysis, BNP= B-type natriuretic peptide, ECW= extracellular water, H=height, ICW=intracellular water, MF-BIA= multiple frequency BIA, N/A = data not available, N= total population, n=heart failure patients, PA= phase angle, Rz/H =resistance/height, SD = standard deviation, SF-BIA= single frequency BIA, TBW=total body water, Xc/H=reactance/height, WRF= worsening renal function

Supplementary information

Supplement3: the uses of BIA and BIVA to assess nutritional status indicating tools, aims and results of study, recruiting patients with cardiac devices

Author	Population	Tools<br>(type of BIA; country made; BIA frequency)	Aims of study relate to the use of BIA or BIVA	Results	Include patients with CIED
Oreopoulos et al. (2010)[32]	Chronic heart failure patients (n=140)	- DEXA,<br>- SF-BIA (Tanita BC544 Ironman scale) - NIR	To compare DEXA and NIR, DEXA and BIA	- Both SF-BIA and NIR show imprecise results of lean body mass and body fat compared to DEXA	No
Castillo-Martínez et al. (2012)[33]	Chronic heart failure patients (n=519)	-MF-BIA (BodyStat QuadScan 4000; Bodystat; 5, 50, 100, and 200 kHz)	To identify cachexia using BIVA	- BIVA can be used to identify cachexia	N/A
Alves et al. (2014)[34]	Chronic heart failure patients (n=55)	DEXA, SF-BIA, MF-BIA<br>- SF-BIA (Bodystat 1500; 50kHz)<br>- MF-BIA (InBody230; 20, 100 kHz)	To compare accuracy of SF-BIA and MF-BIA to DEXA to detect fat mass, fat-free mass index	- DEXA and MF-BIA showed no differences in means of fat mass, fat free mass, and have better agreement than SF-BIA.<br>- SF-BIA show lower estimate for fat free-mass than DEXA and higher bias than MF-BIA in agreement.<br>- Higher correlation with DEXA for MF-BIA than SF-BIA.	No
Sobieszek et al. (2019) [35]	Chronic heart failure patients (n=100)	-MF-BIA (ImpediMed bioimpedance analysis SFB7 Biolmp v1.55; PinkenbaQld, Australia;5, 50, 200 kHz)	To examine electrical parameters in patients with chronic heart failure and the correlation between BIA parameters and the inflammatory status.	-only male participants that the negative correlation between C-reactive protein and BIA parameters, such as PA and reactance, showed ($p<0.01$).<br>- PA and Xc was significantly lower in group with NYHA III-IV than NYHA I-II ($p=0.01$ and $p<0.01$, respectively).	No
González-Islas et al. (2020)[36]	Chronic heart failure patients (n=343)	-MF-BIA (BodyStat QuadScan 4000; Bodystat; 5, 50, 100, and 200 kHz)	1) To examine the changes of body composition in patients with right heart failure and without by using BIVA 2) To assess the potential risks causing CC using BIVA criteria	- 65 patients developed CC at 537 days (the median follow-up)<br>- The risks factor that cause CC were age, right heart failure, PA<5°, total body water after adjusting confounding factors.	N/A
AUC= area under curve, BIVA= bioelectric impedance vector analysis, BIA= bioelectric impedance analysis, CC=cardiac cachexia, CIED= cardiovascular implantable electronic devices, DEXA= dual-energy X-ray absorptiometry, MF-BIA= multiple frequency BIA, N/A = data not available, N= total population, n=heart failure patients, NIR= near-infrared interactance, PA= phase angle, SF-BIA= single frequency BIA, TBW= total body water, Xc =reactance					

Supplementary information

Supplement3: the uses of BIA and BIVA to assess nutritional status indicating tools, aims and results of study, recruiting patients with cardiac devices

Author	Population	Tools<br>(type of BIA; country made; BIA frequency)	Aims of study relate to the use of BIA or BIVA	Results	Include patients with CIED
Hirose et al. (2020)[37]	CVD hospitalised patients (N=412, n=117)	- MF-BIA (InBody S10; Biospace device; 1, 5, 50, 250, 500, and 1000 kHz)	To detect cachexia, sarcopenia, malnutrition using PA	- Identify sarcopenia, cachexia, and malnutrition<br>- PA in males 4.12°<br>- PA in females shows a low AUC and sensitivity	No
Scicchitano et al. (2020)[38]	Acute and chronic heart failure (n=900)	- SF-BIA (CardioEFG, Akern RJL Systems, Florence, Italy; 50 kHz).	The effect of oedema and nutritional status on PA.	- Low PA was associated with severity of heart failure, peripheral oedema, acute heart failure, females.<br>- PA seems to be more specific to determine fluid status than nutritional status.<br>- PA was not different between patients with and without CIED	Yes
Gulatava et al. (2021) [39]	Chronic heart failure (N= 116, n=86)	- SF-BIA (BIA450, BIODYNAMICS, USA; 50kHz)	To examine the differences of body compositions among patients with different severity of chronic heart failure	- PA was significantly lower in NYHA class IV than class II (4.350 vs 6.762, p<0.046).<br>-Reactance, percent extracellular cell mass, percent lean body mass, percent fat mass, percent intra- and extra-cellular water were significantly different between patients with NYHA class II and IV (p<0.05).	N/A
Shah et al. (2021) [40]	Chronic heart failure (n=120)	- MF-BIA (Tanita MC-180 MA scales, Tanita Europe B. V, The Netherlands)) and DEXA.	Comparing body composition measurements obtained from MF-BIA and DEXA.	- The strong correlations of lean mass, fat mass and body mass between DEXA and MF-BIA were reported 0.95, 0.96, 0.84, respectively.<br>- DEXA provided higher fat mass (mean difference -5.1 kg) and lower lean mass (mean difference 5.5 kg) than MF-BIA.<br>- DEXA and BIA should not be used interchangeably.	No
BIVA= bioelectric impedance vector analysis, BIA= bioelectric impedance analysis, BNP= B-type natriuretic peptide, CIED= cardiovascular implantable electronic devices, ECW= extracellular water, DEXA = dual-energy X-ray absorptiometry, MF-BIA= multiple frequency BIA, N/A = data not available, N= total population, n=heart failure patients, NYHA= New York heart association, PA= phase angle, SF-BIA= single frequency BIA, Rz =resistance, TBW = total body water, Xc =reactance					

Supplementary information

Supplement4: evaluating safety of BIA and BIVA indicating tools, aims and results of study

Author	Population	Tools<br>(type of BIA; country made; BIA frequency)	Aims of study relate to the use of BIA or BIVA	Results
Buch et al. (2012) [41]	Patients with PPM	- MF-BIA (InBody 520 multifrequency; 5, 50, and 500 kHz)	Safety and electrical disturbance	No adverse effects on PPM.<br>*depends on PPM company*
Fabregat-Andrés et al. (2015)[42]	Acute heart failure patients (n=21)	- SF-BIA (CardioEFG TM model; 50 kHz)	To examine the interferences of BIA on CIED	No interferences, no over-sensing, no changes in generators of BIA current on CIED report
Meyer et al. (2017) [43]	Chronic heart failure with ICD or CRTD (n=63)	- MF-BIA (Adhesive electrodes (3M red dots); Nutriguard; 5, 50, 100 kHz)	To examine the interferences of BIA on CRTD and ICD	There were no electromagnetic interferences or artifacts observed during differences in BIA frequencies.
Chabin et al. (2019) [44]	Patients with CIED (n=200)	- MF-BIA (Nutriguard-MS; München, Germany; 5, 50 and 100 kHz)	To examine the interferences of BIA on CIED	Safe for CIED, there were no over- or under- sensing during telemetry monitoring, no interferences between program and devices.
Garlini et al. (2020) [45]	Patients with CIED (n=43)	- SF-BIA (Tetrapolar device Biodynamics 450; Biodynamics Corp., Seattle, Washington, USA; 50 kHz frequency)	To examine the interferences of BIA on CIED	Safe for CIED and no malfunction
Roehrich et al. (2020)[46]	Advance heart failure patients with CIED (n=217)<br>*20% of patients had no CIED*	- MF-BIA (N/A; 5, 50, 100 kHz)	To examine the safety of BIA in patients who are susceptible to have arrythmia and/or implanted CIED or continuous-flow ventricular assist device	There was no malfunctions and interferences of CIED and continuous-flow ventricular assist device while using BIA and after using BIA in 30 minutes in all patients regardless the version of devices and device manufactures.
BIVA= bioelectric impedance vector analysis, BIA= bioelectric impedance analysis, CIED= cardiovascular implantable electronic devices, MF-BIA= multiple frequency BIA, N= total population, n=heart failure patients, SF-BIA= single frequency BIA				

Supplementary information

References:

[1] Colin-Ramirez E, Castillo-Martinez L, Orea-Tejeda A, Asensio Lafuente E, Torres Villanueva F, Rebollar Gonzalez V, et al. Body composition and echocardiographic abnormalities associated to anemia and volume overload in heart failure patients. Clin Nutr. 2006;25:746-57.

[2] Castillo Martinez L, Colin Ramirez E, Orea Tejeda A, Asensio Lafuente E, Bernal Rosales LP, Rebollar Gonzalez V, et al. Bioelectrical impedance and strength measurements in patients with heart failure: comparison with functional class. Nutrition. 2007;23:412-8.

[3] Parrinello G, Paterna S, Di Pasquale P, Torres D, Fatta A, Mezzero M, et al. The Usefulness of Bioelectrical Impedance Analysis in Differentiating Dyspnea Due to Decompensated Heart Failure. Journal of Cardiac Failure. 2008;14:676-86.

[4] Di Somma S, De Berardinis B, Bongiovanni C, Marino R, Ferri E, Alfei B. Use of BNP and Bioimpedance to Drive Therapy in Heart Failure Patients. Congestive Heart Failure. 2010;16:S56-S61.

[5] Valle R, Aspromonte N, Milani L, Peacock FW, Maisel AS, Santini M, et al. Optimizing fluid management in patients with acute decompensated heart failure (ADHF): the emerging role of combined measurement of body hydration status and brain natriuretic peptide (BNP) levels. Heart Fail Rev. 2011;16:519-29.

[6] Di Somma S, Lalle I, Magrini L, Russo V, Navarin S, Castello L, et al. Additive diagnostic and prognostic value of bioelectrical impedance vector analysis (BIVA) to brain natriuretic peptide 'grey-zone' in patients with acute heart failure in the emergency department. Eur Heart J Acute Cardiovasc Care. 2014;3:167-75.

[7] Alves FD, Souza GC, Aliti GB, Rabelo-Silva ER, Clausell N, Biolo A. Dynamic changes in bioelectrical impedance vector analysis and phase angle in acute decompensated heart failure. Nutrition. 2015;31:84-9.

[8] Génot N, Mewton N, Bresson D, Zouaghi O, Francois L, Delwarde B, et al. Bioelectrical impedance analysis for heart failure diagnosis in the ED. The American Journal of Emergency Medicine. 2015;33:1025-9.

[9] Sakaguchi T, Yasumura K, Nishida H, Inoue H, Furukawa T, Shinouchi K, et al. Quantitative Assessment of Fluid Accumulation Using Bioelectrical Impedance Analysis in Patients With Acute Decompensated Heart Failure. Circ J. 2015;79:2616-22.

[10] Yamazoe M, Mizuno A, Niwa K, Isobe M. Edema index measured by bioelectrical impedance analysis as a predictor of fluid reduction needed to remove clinical congestion in acute heart failure. International Journal of Cardiology. 2015;201:190-2.

[11] Gil Martínez P, Mesado Martínez D, Curbelo García J, Cadiñanos Loidi J. Amino-terminal pro–B-type natriuretic peptide, inferior vena cava ultrasound, and biolectrical impedance analysis for the diagnosis of acute decompensated CHF. The American Journal of Emergency Medicine. 2016;34:1817-22.

[12] Massari F, Iacoviello M, Scicchitano P, Mastropasqua F, Guida P, Riccioni G, et al. Accuracy of bioimpedance vector analysis and brain natriuretic peptide in detection of peripheral edema in acute and chronic heart failure. Heart Lung. 2016;45:319-26.

[13] Santarelli S, Russo V, Lalle I, De Berardinis B, Navarin S, Magrini L, et al. Usefulness of combining admission brain natriuretic peptide (BNP) plus hospital discharge bioelectrical impedance vector analysis (BIVA) in predicting 90 days cardiovascular mortality in patients with acute heart failure. Intern Emerg Med. 2017;12:445-51.

[14] Santarelli S, Russo V, Lalle I, De Berardinis B, Vetrone F, Magrini L, et al. Prognostic value of decreased peripheral congestion detected by Bioelectrical Impedance Vector Analysis (BIVA) in patients hospitalized for acute heart failure: BIVA prognostic value in acute heart failure. European Heart Journal Acute Cardiovascular Care. 2017;6:339-47.

[15] De Ieso F, Mutke MR, Brasier NK, Raichle CJ, Keller B, Sucker C, et al. Body composition analysis in patients with acute heart failure: the Scale Heart Failure trial. ESC heart failure. 2021;8:4593-606.

[16] Marawan A, Thomas GK, Canada JM, Billingsley HE, Dixon DL, Van Tassell BW, et al. Edema Index Predicts Cardiorespiratory Fitness in Patients With Heart Failure With Reduced Ejection Fraction and Type 2 Diabetes Mellitus. J Am Heart Assoc. 2021;10:e018631-e.

[17] Colín-Ramírez E, Castillo-Martínez L, Orea-Tejeda A, Vázquez-Durán M, Rodríguez AE, Keirns-Davis C. Bioelectrical impedance phase angle as a prognostic marker in chronic heart failure. Nutrition. 2012;28:901-5.

[18] Liu MH, Wang CH, Huang YY, Tung TH, Lee CM, Yang NI, et al. Edema index established by a segmental multifrequency bioelectrical impedance analysis provides prognostic value in acute heart failure. J Cardiovasc Med (Hagerstown). 2012;13:299-306.

[19] Liu MH, Wang CH, Huang YY, Tung TH, Lee CM, Yang NI, et al. Edema index-guided disease management improves 6-month outcomes of patients with acute heart failure. Int Heart J. 2012;53:11-7.

[20] De Berardinis B, Magrini L, Zampini G, Zancla B, Salerno G, Cardelli P, et al. Usefulness of combining galectin-3 and BIVA assessments in predicting short- and long-term events in patients admitted for acute heart failure. Biomed Res Int. 2014;2014:983098.

[21] Alves FD, Souza GC, Clausell N, Biolo A. Prognostic role of phase angle in hospitalized patients with acute decompensated heart failure. Clinical Nutrition. 2016;35:1530-4.

[22] Núñez J, Mascarell B, Stubbe H, Ventura S, Bonanad C, Bodí V, et al. Bioelectrical impedance vector analysis and clinical outcomes in patients with acute heart failure. J Cardiovasc Med (Hagerstown). 2016;17:283-90.

[23] Lyons KJ, Bischoff MK, Fonarow GC, Horwich TB. Noninvasive Bioelectrical Impedance for Predicting Clinical Outcomes in Outpatients With Heart Failure. Crit Pathw Cardiol. 2017;16:32-6.

[24] Curbelo J, Rodriguez-Cortes P, Aguilera M, Gil-Martinez P, Martín D, Suarez Fernandez C. Comparison between inferior vena cava ultrasound, lung ultrasound, bioelectric impedance analysis, and natriuretic peptides in chronic heart failure. Current Medical Research and Opinion. 2019;35:705-13.

[25] Park CS, Lee SE, Cho HJ, Kim YJ, Kang HJ, Oh BH, et al. Body fluid status assessment by bio-impedance analysis in patients presenting to the emergency department with dyspnea. Korean J Intern Med. 2018;33:911-21.

[26] Thomas E, Gupta PP, Fonarow GC, Horwich TB. Bioelectrical impedance analysis of body composition and survival in patients with heart failure. Clin Cardiol. 2019;42:129-35.

[27] Massari F, Scicchitano P, Ciccone MM, Caldarola P, Aspromonte N, Iacoviello M, et al. Bioimpedance vector analysis predicts hospital length of stay in acute heart failure. Nutrition. 2019;61:56-60.
[28] Castillo-Martínez L, Rodríguez-García WD, González-Islas DG, Orea-Tejeda A, Lozada-Mellado M, Rodríguez-Silverio J, et al. Abnormal fluid distribution and low handgrip strength index as predictors of mortality in Mexican patients with chronic heart failure. Nutrition. 2020;72:110699.
[29] Massari F, Scicchitano P, Iacoviello M, Passantino A, Guida P, Sanasi M, et al. Multiparametric approach to congestion for predicting long-term survival in heart failure. Journal of Cardiology. 2020;75:47-52.
[30] Kammar-García A, Castillo-Martínez L, Villanueva-Juárez JL, Pérez-Pérez A, Rocha-González HI, Arrieta-Valencia J, et al. Comparison of Bioelectrical Impedance Analysis Parameters for the Detection of Fluid Overload in the Prediction of Mortality in Patients Admitted at the Emergency Department. JPEN J Parenter Enteral Nutr. 2021;45:414-22.
[31] Villacorta H, Villacorta AS, Villacorta LSdC, Xavier AR, Kanaan S, Rohen FM, et al. Worsening Renal Function and Congestion in Patients with Acute Heart Failure: A Study with Bioelectrical Impedance Vector Analysis (BIVA) and Neutrophil Gelatinase-Associated Lipocalin (NGAL). Arquivos Brasileiros de Cardiologia. 2021;116:715-24.
[32] Oreopoulos A, Kalantar-Zadeh K, McAlister FA, Ezekowitz JA, Fonarow GC, Johnson JA, et al. Comparison of direct body composition assessment methods in patients with chronic heart failure. J Card Fail. 2010;16:867-72.
[33] Castillo-Martínez L, Colín-Ramírez E, Orea-Tejeda A, González Islas DG, Rodríguez García WD, Santillán Díaz C, et al. Cachexia assessed by bioimpedance vector analysis as a prognostic indicator in chronic stable heart failure patients. Nutrition. 2012;28:886-91.
[34] Alves FD, Souza GC, Biolo A, Clausell N. Comparison of two bioelectrical impedance devices and dual-energy X-ray absorptiometry to evaluate body composition in heart failure. J Hum Nutr Diet. 2014;27:632-8.

[35] Sobieszek G, Mlak R, Skwarek-Dziekanowska A, Jurzak-Myśliwy A, Homa-Mlak I, Małecka-Massalska T. Electrical Changes in Polish Patients with Chronic Heart Failure: Preliminary Observations. Medicina (Kaunas). 2019;55:484.

[36] González-Islas D, Arámbula-Garza E, Orea-Tejeda A, Castillo-Martínez L, Keirns-Davies C, Salgado-Fernández F, et al. Body composition changes assessment by bioelectrical impedance vectorial analysis in right heart failure and left heart failure. Heart & Lung. 2020;49:42-7.

[37] Hirose S, Nakajima T, Nozawa N, Katayanagi S, Ishizaka H, Mizushima Y, et al. Phase Angle as an Indicator of Sarcopenia, Malnutrition, and Cachexia in Inpatients with Cardiovascular Diseases. J Clin Med. 2020;9.

[38] Scicchitano P, Ciccone MM, Passantino A, Valle R, De Palo M, Sasanelli P, et al. Congestion and nutrition as determinants of bioelectrical phase angle in heart failure. Heart & Lung. 2020;49:724-8.

[39] Gulatava N, Tabagari N, Tabagari S. BIOELECTRICAL IMPENDANCE ANALYSIS OF BODY COMPOSITION IN PATIENTS WITH CHRONIC HEART FAILURE. Georgian Med News. 2021:94-8.

[40] Shah P, Abel AAI, Boyalla V, Pellicori P, Kallvikbacka-Bennett A, Sze S, et al. A comparison of non-invasive methods of measuring body composition in patients with heart failure: a report from SICA-HF. ESC Heart Failure. 2021;8:3929-34.

[41] Buch E, Bradfield J, Larson T, Horwich T. Effect of bioimpedance body composition analysis on function of implanted cardiac devices. Pacing Clin Electrophysiol. 2012;35:681-4.

[42] Fabregat-Andrés Ó, Fácila L, Montagud-Balaguer V, Galán-Serrano A. Systemic bioimpedance analysis in patients with implanted cardiac stimulation devices. Nefrología (English Edition). 2015;35:345-6.

[43] Meyer P, Makhlouf AM, Mondouagne Engkolo LP, Trentaz F, Thibault R, Pichard C, et al. Safety of Bioelectrical Impedance Analysis in Patients Equipped With Implantable Cardioverter Defibrillators. JPEN J Parenter Enteral Nutr. 2017;41:981-5.

[44] Chabin X, Taghli-Lamallem O, Mulliez A, Bordachar P, Jean F, Futier E, et al. Bioimpedance analysis is safe in patients with implanted cardiac electronic devices. Clin Nutr. 2019;38:806-11.

[45] Garlini LM, Alves FD, Kochi A, Zuchinali P, Zimerman L, Pimentel M, et al. Safety and Results of Bioelectrical Impedance Analysis in Patients with Cardiac Implantable Electronic Devices. Braz J Cardiovasc Surg. 2020;35:169-74.

[46] Roehrich L, Suendermann S, Just IA, Knierim J, Mulzer J, Mueller M, et al. Safety of bioelectrical impedance analysis in advanced heart failure patients. Pacing and Clinical Electrophysiology. 2020;43:1078-85.
